# Chitosan/POSS Hybrid Hydrogels for Bone Tissue Engineering

**DOI:** 10.3390/ma15228208

**Published:** 2022-11-18

**Authors:** Consuelo Celesti, Daniela Iannazzo, Claudia Espro, Annamaria Visco, Laura Legnani, Lucia Veltri, Giuseppa Visalli, Angela Di Pietro, Paola Bottino, Maria Assunta Chiacchio

**Affiliations:** 1 Department of Engineering, University of Messina, Contrada Di Dio, 98166 Messina, Italy; 2 Department of Clinical and Experimental Medicine, University of Messina, Via Consolare Valeria, 98125 Messina, Italy; 3 Institute for Polymers, Composites and Biomaterials, National Research Council CNR, Via P. Gaifami 18, 95126 Catania, Italy; 4 Department of Biotechnology and Biosciences, University of Milano-Bicocca, Piazza della Scienza 2, 20126 Milano, Italy; 5 Department of Chemistry and Chemical Technologies, University of Calabria, Via Pietro Bucci 12/C, 87036 Aracavacata di Rende, Italy; 6 Department of Biomedical and Dental Sciences and Morphological and Functional Images, University Hospital of Messina, Via Consolare Valeria, 1, 98100 Messina, Italy; 7 Department of Drug and Health Sciences, University of Catania, Viale A. Doria 6, 95125 Catania, Italy

**Keywords:** biomaterials, hydrogels, tissue engineering, hybrid materials, biopolymers, polyhedral oligomeric silsesquioxanes

## Abstract

Hybrid hydrogels composed of chitosan (CS) have shown great potential in bone tissue engineering and regeneration. The introduction of polyhedral oligomeric silsesquioxanes (POSS) in the biopolymeric matrix has been demonstrated to improve the rheological and biological properties of the hybrid composites. In this work, we have integrated the favourable features of chitosan (CS) and POSS nanoparticles to design new nanocomposites for bone tissue regeneration, focusing our attention on the effect of POSS concentration within the CS matrix (0.5, 1, and 1.5 equivalents in weight of POSS with respect to CS) on the chemical, physical, rheological, and in vitro biological properties of the final composites. The drug release ability of the synthesized hydrogel scaffolds were also investigated using, as the model drug, ketoprofen, that was included in the scaffold during the gelling procedure, showing a more controlled release for the hybrids with respect to CS (86–91% of drug released after two weeks). The results of the in vitro biological tests performed on human fetal osteoblastic cells (hFOB 1.19) culture demonstrated the great biocompatibility of the hybrid materials. The hybrids, at the different POSS concentrations, showed values of cell mortality superimposable with control cells (11.1 vs. 9.8%), thus revealing the CS/POSS hydrogels as possible candidates for bone tissue engineering applications.

## 1. Introduction

The treatment of bone defects in dental and orthopaedic surgery represents a major medical and socio-economic challenge [1]. Bone tissue engineering has shown the potential to overcome some limitations of conventional bone grafts, aiming to prompt functional bone regeneration through the synergistic combination of advanced biomaterials, stem cells, and biofactor therapy [2,3]. In this context, biocompatible and biomimetic, natural, or synthetic materials able to encapsulate cells and induce the natural formation of bone tissue can provide a very important tool in treating these defects [4,5]. The main challenge in bone tissue engineering today is the development of biomaterials and biocomposites for integration in the body and able to promote the exchange of nutrients with human environments [6,7]. All these specific functional features can be found in composites based on natural polymers, containing inorganic materials capable of mimicking the natural structure of bone tissue, and lead to the generation of bone scaffolds to be employed both as bone substitutes and as drug delivery systems [8,9,10]. Among the different biocompatible biopolymers, chitosan (CS) has been widely investigated in various fields of science, and potential applications in tissue engineering are constantly growing [11]. This biopolymer, a linear deacetylated polysaccharide found in high abundance in the exoskeleton of crustaceans, is useful for tissue regeneration because of its ability to rapidly coagulate blood, promote healing, and produce hypoallergenic response because of its biocompatibility, biodegradability, and nontoxicity [12]. In addition, upon gelling, and using cross-linking reagents such as genipin or glutaraldehyde, it can take a desired 3D shape [13]. However, besides these interesting features, the applications of hydrogels based on this natural polysaccharide are hindered by the low mechanical strength, burst drug release, and fast hydrolysis, which limit its use for bone tissue engineering applications [13,14,15]. An approach to improve the mechanical properties and the biological features of chitosan-based scaffolds is usually to reinforce the hydrogels with bioactive ceramic materials, such as silica nanoparticles, hydroxyapatite (HA), bioglass ceramics, zirconium oxide, and titanium dioxide [16,17,18,19,20]. Polyhedral oligomeric silsesquioxanes (POSS), a class of nanostructured silica-based compounds, have been extensively investigated for the development of composite materials able to induce bone regeneration, as well as to serve as drug delivery agents [21,22]. These hybrid inorganic-organic molecules with a cage-like structure, whose repeating unit has the formula RSiO_3__/__2_ and size in the range of 1–3 nm, can be properly functionalized and covalently linked to polymers to improve their mechanical, rheological, and biological properties [23,24,25,26,27,28]. POSS nanostructures are resorbable, bioactive, and biocompatible; in vitro biological assays have shown that POSS molecules encourage bone stroma cell proliferation, differentiation, and apatite deposition [21,26]. Biocompatible polyvinyl acetate/POSS composites have been proven to enhance bone regeneration, improve apatite layer formation at the hydrogel surface, and guide cell adhesion and diffusion [25]. The incorporation of octa(tetramethylammonium)-POSS into the chitosan matrix has also been shown to increase the plasma protein adsorption on the material surface and to enhance the surface roughness, protein adsorption, and swelling ability of the composite [23,29]. Moreover, CS-POSS scaffolds have been demonstrated to increase alkaline phosphatase activity, octeocalcin secretion, and biomineralization of cells, further highlighting these composites as promising bioactive agents for bone tissue engineering [30].

**Figure 1:**
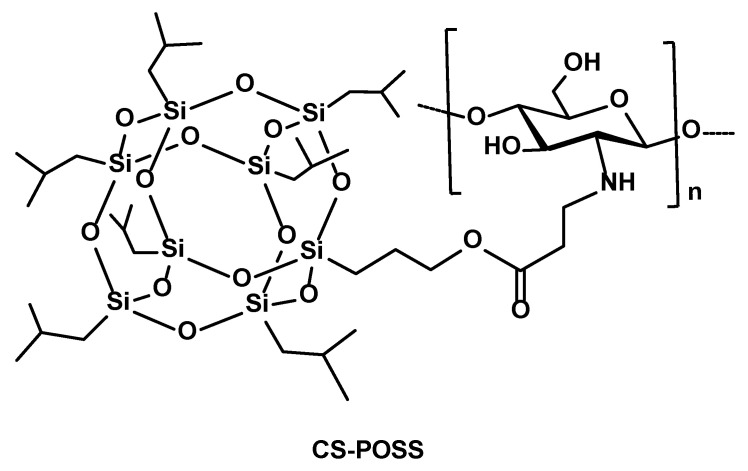
Structure of CS-POSS.

Considering the reported interesting properties of CS/POSS hybrids for tissue engineering applications, the aim of this study was to investigate, for the first time, the effect of POSS concentration within the CS matrix on the chemical, physical, mechanical, and in vitro biological properties of the composites. To achieve this, we took advantage of the presence of an unsaturated ester group present in a heptaisobutyl POSS structure to covalently bind it to chitosan. The Michael-type addition of the NH_2_ group of chitosan to the double bond present in the POSS structure was performed using POSS amounts of 0.5, 1, and 1.5 wt%, with respect to chitosan, thus allowing the formation of CS-POSS hybrid materials with different POSS contents (Figure 1).

The subsequent reaction with genipin as the cross-linking agent, a natural compound endowed with low toxicity [29], allowed the formation of the corresponding hydrogels, which were investigated by chemical, physical, morphological, and rheological characterizations. The drug release ability of the synthesized hydrogel scaffolds was also investigated using, as a model drug, ketoprofen, that was inserted into the scaffold during the gelling procedure. The results of in vitro biological tests performed on human fetal osteoblastic cells culture (hFOB 1.19) demonstrated the great biocompatibility of the hybrid materials which, even at the highest concentrations of POSS within the CS matrix, exhibited cells growth analogous to the control, thus demonstrating CS/POSS hydrogels as possible candidates for tissue engineering applications.

## 2. Materials and Methods

### 2.1. Materials

The reagents chitosan (medium molecular weight, deacetylation 75–85%), acetic acid (99.9%), and ketoprofen (>99%), ninhydrin, were purchased from Sigma Aldrich (St. Louis, MO, USA); acryloxypropyl-heptaisobutyl-POSS (MA0701, C34H72O14Si8, MW: 929.61 g/mol) was purchased from Hybrid Plastics (USA); genipin (>98%) was acquired from Carbosynth (St. Gallen, Switzerland). All chemicals and solvents were used without further purification. Cell line hFOB 1.19 was purchased from ATCC-(LGC Standards, Milano, Italia), while fluorophores and cell culture media were purchased from Sigma-Aldrich. The infrared spectra were obtained using a Two FT-IR Spectrometer (PerkinElmer Inc.), by the ATR method in the range of 4000–500 cm^−1^. Thermogravimetric (TGA) characterizations were performed using a TAQ500 instrument (TA Instruments, New Castle, DE, USA) from 20 °C to 600 °C, at a rate of 20 °C/min, under inert atmosphere. Rheological measurements were accomplished using a rotational rheometer (Mod. SR5, Rheometric scientific, Piscataway, NJ, USA) at 37 °C, with parallel-plate geometry. UV analyses were carried out with a Thermo Nicolet mod. Evolution 500 spectrophotometer, by measuring the absorbance of the drug at 260 nm. Fluorimetric analyses were performed using a microtiter plate reader (Tecan Italia, Milan, Italy); for the morphological analyses, a TCS SP2 instrument (Leica Microsystems, Heidelberg, Germany) equipped with an Ar/Kr laser and coupled with a microscope (Leica DM IRB) was employed.

### 2.2. Methods

#### 2.2.1. Synthesis of CS-POSS Hybrids

CS-POSS hybrids were synthesized by Michael type reaction [31]. Briefly, chitosan powder (200 mg) was dissolved in 2% aqueous acetic acid solution for 30 min at 45 °C; after that, the dispersion was treated with the three different amounts of acryloxypropyl-heptaisobutyl-POSS; i.e., 100 mg (0.5 equiv, 0.11 mmol), 200 mg (1 equiv, 0.21 mmol), and 300 mg (1.5 equiv, 0.32 mmol). The reaction mixture was left under magnetic stirring, at reflux (50 °C) overnight. The obtained samples, CS-POSS **1** (ratio CS/POSS = 1:0.5), CS-POSS **2** (ratio CS/POSS = 1:1), and CS-POSS **3** (ratio CS/POSS = 1:1.5), were treated with a saturated solution of NaHCO_3_ until neutral pH was reached, and subsequently, purified through dialysis bags (MW of 12,000 Da) for two days. Finally, the samples were lyophilized by freeze-drying at −80 °C for 72 h and used for subsequent characterizations. The amount of free amino groups present in the CS and in the CS-POSS hybrids were evaluated by UV–vis absorption spectra, after reaction with ninhydrin, following a method reported in the literature [32]. Briefly, CS- or CS-POSS-based samples dispersed in 2% aqueous acetic acid (0.1 mg/mL) were added with ninhydrin (2 mg/mL), placed in a boiling water bath and left under stirring for 20 min. After cooling, the absorbances of the solutions were measured at 570 nm.

#### 2.2.2. Synthesis of CS and CS-POSS Hybrid Hydrogels

Chitosan powder (200 mg) or CS-POSS hybrid samples (200 mg) were dispersed in a 2% aqueous acetic acid solution for 30 min, at 45 °C. Then, 20 mg (0.1 mmol) of genipin was slowly added to the mixture leading to the formation of a 3D gel. The hydrogels were then rinsed with deionized water and then stored at 15 °C in a hermetic sealed pan with a constant relative humidity. The amount of water content, measured by drying the hydrogels in a beaker for 24 h at 37 °C and at a vacuum drying pressure of 65 mbar until constant weight, was found to be of 94 wt%, 82 wt%, 78 wt%, and 73 wt% for CS, CS-POSS **1**, CS-POSS **2**, and CS-POSS **3** hydrogels, respectively.

#### 2.2.3. Preparation of Ketoprofen-Doped CS and CS-POSS Hydrogels

Ketoprofen-doped CS and CS-POSS hydrogels were prepared and stored, following the procedure described in Section 2.2.2. The drug ketoprofen as lysine salt (160 mg, 0.67 mmol) was dissolved in deionized water (2 mL) and then added to the mixture before the genipin addition step.

#### 2.2.4. Drug Release Studies

The drug release behavior was evaluated from ketoprofen-doped CS and CS-POSS hydrogels in order to study the effect of the presence of the silica cage on the drug release properties of chitosan-based matrices. The study was conducted at a temperature of 37 °C, in phosphate buffer solution (pH 7.4) using the dialysis bag diffusion technique. The amount of drug released was evaluated by UV-Vis absorption analyses, measuring the absorbance of the drug at 260 nm, referring to a calibration curve recorded under the same conditions. The Linearity Standard calibration curve was found to be linear at concentrations ranging from 0.25 to 250 ng/mL (y = 0.0854x + 0.0357) with a correlation coefficient of 0.9772.

#### 2.2.5. Rheological Studies

Rheological analyses were carried out on cylindrical samples (diameter of 25 mm and height of 1 mm) by means of a rotational rheometer. Measurements were carried out in a controlled humidity environment by filling the specimen holding system with deionized water and sealed with an insulating cover to avoid drying of the samples during the tests [33]. The dynamic stress sweep test (frequency of 1 Hz) was performed in the stress range of 0.5 Pa-1000 Pa in order to evaluate the linear viscoelastic region (LVR) [34]. Frequency sweep tests (in stress control) were accomplished in the frequency range of 0.01–200 rad/s at a constant stress (10 Pa). Rheological properties, namely the frequency response of G′ and the complex viscosity η*, were monitored 30 min after the start of crosslinking. Each test was performed in duplicate.

#### 2.2.6. Cell Cultures and Biological Assays

Human fetal osteoblastic cells (hFOB 1.19; ATCC-CRL-11372™) were used as in vitro models to assess the biocompatibility of the synthesized CS- and CS-POSS-based hydrogels. The cells were grown in a 1:1 mixture of Ham’s F12 and DMEM medium without phenol red and with the addition of 2.5 mM L-glutamine, 0.3 mg/mL G418, and 10% of fetal bovine serum, at 34 °C in an atmosphere of 5% CO_2_, in order to mimic the natural living tissue environment. The samples (100 μL) were gelified at room temperature in 6-well cell culture plates, to which the cell suspension (5 × 10^4^ cells/mL) was added while cells, cultured in their specific medium and grown in wells without the gelified sample, were used as negative controls. In each well, the area covered by the hydrogels was calculated using ImageJ software (imagej.nih.gov/ij/index.html, accessed on 8 September 2022) in order to use only wells with a sample area equal to 35% (±5) of the entire well area. Cells were grown on the formed hydrogels for 48 h. The biocompatibility of the tested materials with increasing amounts of POSS concentrations (0 wt%, 0.5 wt%, 1.0 wt%, and 1.5% wt%) was evaluated by fluorimetric analysis (λ exc 493–λ em 636 nm) using a DNA intercalating probe propidium iodine (PI 20 μg/mL) for 15 min at −20 °C. The analyses were performed at least in triplicate both on the cell medium to detect the detached cells (death cells) and, after cell permeabilization by methanol, on the adherent cells to assess the viable cells. The data were presented as the mean ± standard deviation (SD) and were analysed by one-way analysis of variance (ANOVA) to assess possible significant differences, which were accepted at *p* < 0.05 (GraphPad Prism 8). To evaluate any morphological alterations due to the effects of the hydrogels, confocal microscope observations were performed by a CLSM equipped with a 40× 1.0 NA immersion objective and a TCS SP2 instrument (Leica Microsystem Heidelberg, Mannaheim, Germany). The cell-permeable metachromatic fluorophore acrydine orange (AO) was used. AO allows you to clearly visualize both the cytoplasmic compartment where, thanks to the presence of the acid compartment, the organelles that emit red fluorescence can be seen, and the nuclear ones with green fluorescence, due to the bond between AO in monomeric form and DNA double helix.

## 3. Results and Discussion

### 3.1. Synthesis of CS-POSS Hydrogels

**Scheme 1:**
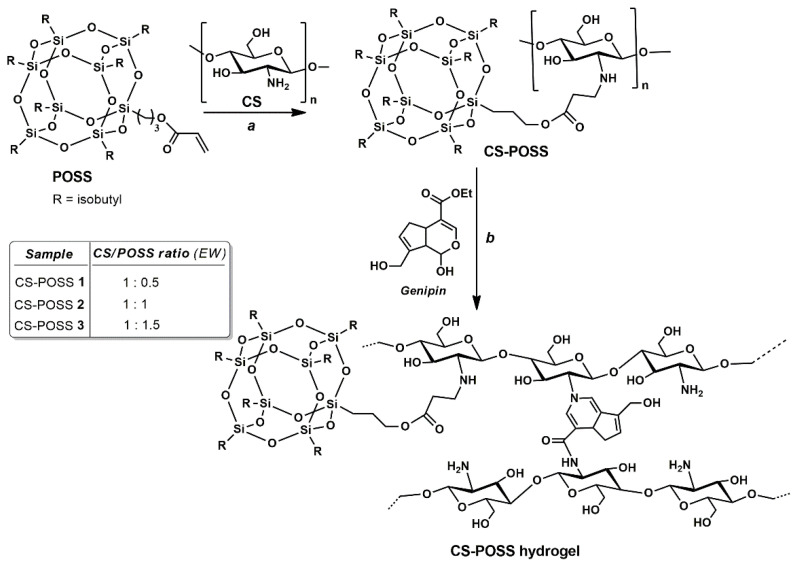
Synthesis of CS-POSS hybrid hydrogels. Reagents and conditions: (**a**) acetic acid 2%, 50 °C, 15 h, 50 °C, overnight, then NaHCO_3_ until pH 7. (**b**) genipin, acetic acid 2%, 45 °C, 30 min.

The first step to obtain CS-POSS-based hydrogels was to perform the Michael addition reaction between the amino group of chitosan and the terminal double bond present in the POSS molecule. In order to investigate how a different amount of POSS can tune the chemical-physical, mechanical, morphological, and biological behaviour of the composites, we used 0.5, 1, and 1.5 equivalents in weight of POSS with respect to the used weight of CS. The reaction, performed in water solution and at a mild temperature (50 °C) afforded the conjugated compounds, which were purified through a dialysis bag able to retain compounds with MW of 12,000 or higher. The subsequent reaction with the cross-linking agent genipin allows the binding of two amine groups between the neighbouring chains of the CS polymer, leading to the formation of the corresponding hydrogels (Scheme 1). The amount of free amino groups available for the cross-linking reaction of the hybrid materials at different CS/POSS ratios was spectrophotometrically quantified by measuring the absorbances of the samples at 570 nm, after reaction with ninhydrin, as described in Section 2.2.1 [32]. As expected, the amount of these groups decreased from 82% in the starting CS to 67% for CS-POSS **1**, 53% to CS-POSS **2**, and to 39% to CS-POSS **3**.

**Figure 2:**
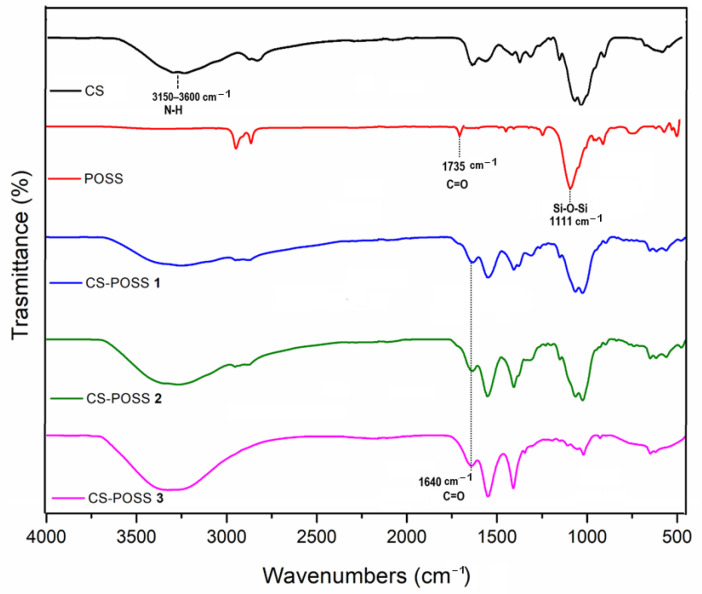
FTIR spectra of samples CS, POSS, CS-POSS **1**, CS-POSS **2**, and CS-POSS **3**.

The effectiveness of Michael type reactions in affording new hybrid materials after covalent binding was investigated by FTIR spectroscopy and TGA analyses for all the synthesized samples, before the gelation procedures. FTIR spectra demonstrated the presence of a covalent bond between CS and POSS, at the different concentrations (Figure 2). The FTIR spectrum of chitosan shows an intense band at 3150–3600 cm^−1^ due to the stretching of N–H and O–H bonds, a band at 1580 cm^−1^ attributable to N–H bending, and a peak at 1028 cm^−1^ corresponding to the stretching of the C–O group; in addition, the presence of the peak at 1650 cm^−1^, corresponding to C=O stretching of the primary amide, confirms the existence of residual N-acetyl groups in the chitosan structure. The POSS sample shows the diagnostic signals at 1111 cm^−1^ attributable to Si-O-Si stretching of the silica cage, and the peak at 1735 cm^−1^ corresponding to C=O stretching of the ester group. In the spectra of the three CS-POSS conjugate samples, namely CS-POSS **1**, CS-POSS **2**, and CS-POSS **3**, containing 0.5, 1, and 1.5 equivalents of POSS in weight, respectively, the stretching frequency of the C=O ester groups occurred at lower frequencies (~1640 cm^−1^) than that observed for the starting POSS (1735 cm^−1^). The observed blue shift is attributable to the weakening of the C=O ester bond strength, since the double bond of the α,β-unsaturated ester is no longer present in the hybrid systems [35,36], thus confirming the covalent bond between the CS and POSS molecules. Furthermore, the other characteristic peaks of chitosan and POSS were observed in the FT-IR spectra of all synthesized samples.

**Figure 3:**
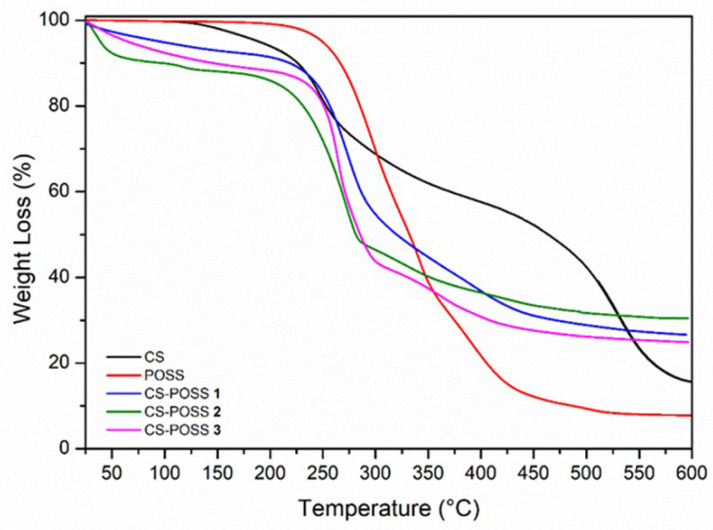
TGA curves for CS-, POSS-, and CS-POSS-based samples, CS-POSS **1**, CS-POSS **2**, and CS-POSS **3**. All experiments were performed in triplicate under argon atmosphere.

The conjugation between chitosan with POSS leading to CS-POSS samples was confirmed also by TGA, performed under inert atmosphere (Figure 3). All the samples were heated up to 600 °C under argon flow, at a rate of 20 °C/min. The CS sample shows important weight loss in the range of 200–350 °C and 400–600 °C, with a 70% total weight loss. The POSS sample exhibits an initial thermal decomposition temperature at 235 °C, a maximum temperature of decomposition at 293 °C, and complete thermal degradation at 600 °C. The TGA curves of the CS-POSS hybrid materials at different concentrations show different profiles with respect to what was observed for chitosan and POSS precursors. For these samples, three different stages of weight loss can be observed: the first at 180–250 °C and the second at 280–400 °C, with a total weight loss of about 75%. The different profiles of the hybrid materials, CS-POSS **1**, CS-POSS **2**, and CS-POSS **3**, are indicative of their different chemical composition with respect to CS and POSS precursors after the conjugation reactions, differing only in the amount of POSS molecules inside the hybrids, as also reported in the literature for similar systems [24,27]. These results, repeated in triplicate under identical experimental conditions, showed consistent precision and repeatability, with very low standard deviations (SD ≤ 0.55).

**Figure 4:**
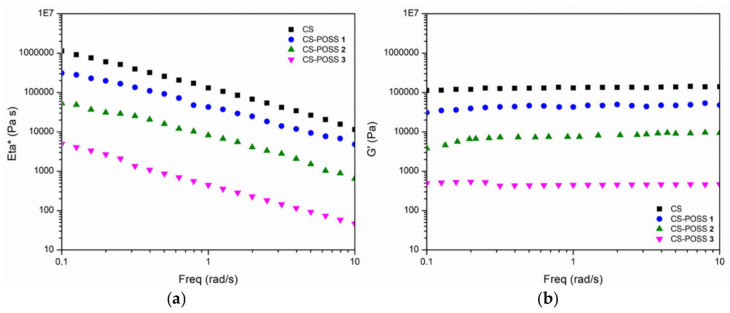
Complex viscosity (η*) (**a**) and G′ modulus (**b**) vs. frequency for CS and CS-POSS samples, at different POSS concentrations.

All the hybrid samples and CS were then treated with genipin which, after cross-linking reaction with the free amino groups present in the CS, gave the corresponding hydrogels. The rheological properties of the so synthesized hydrogels were evaluated in dynamic stress and frequency scan modes to verify the linear viscoelastic region (LVR) and to evaluate the main rheological features (i.e., frequency response of G′ and complex viscosity η*), as described in Section 2.2.5. Figure 4 shows the complex viscosity and the elastic modulus of all four samples, as a function of frequency, in the range 0.01–200 rad/s. The general trend is that the viscosity of all materials decreases as the frequency value increases, as expected. At higher frequencies (values greater than ~10 rad/s) the viscosity becomes constant or slightly increases; this is due to the “upper-Newtonian” behavior. Similarly, at viscosity values lower than ~0.1 rad/s, the viscosity is almost constant: this is due to the “lower Newtonian” behavior. From the data in Table 1, we can see that the higher the POSS content, the lower the viscosity and the lower the value of the modulus G′. This occurs at both low (0.1 rad/s) and high (10 rad/s) frequency values. In detail, the G′ value of the CS (at 0.1 rad/s) is 118.183 Pa and the viscosity value is 1.197.866 Pa*s, while in the CS-POSS **3** sample, the values drop three orders of magnitude (478 Pa and 5132 Pa* s, respectively). A similar trend is repeated at a frequency of 10 rad/sec. The rheological results are in agreement with the data obtained from the UV-vis quantification of the free amino groups. In fact, the progressive decrease of these groups needed for the cross-linking reaction with genipin, from CS to CS-POSS **1**, CS-POSS **2**, and CS-POSS **3** samples, leads to a progressive reduction of the reticulation degree. This decrease, together with the steric hindrance induced by the increasing POSS concentrations, also reduces the overall degree of cross-linking of the polymer matrix. Consequently, there is a decrease in stiffness (as suggested by the lowering in the G′ modulus) and in structural complexity (as suggested by the reduction in viscosity). Thus, the presence of the three different concentrations of POSS in the hydrogel changes the rheological response; the steric hindrance of POSS decreases the overall degree of cross-linking of the polymer matrix, decreasing its structural complexity. Thus, the stiffness (and viscosity) of CS-POSS-based samples progressively decreases by increasing the POSS content inside the polymeric matrix, resulting in materials that are more ductile, even more so than pure CS.

### 3.2. Drug Release Studies of CS and CS-POSS Hydrogels

**Figure 5:**
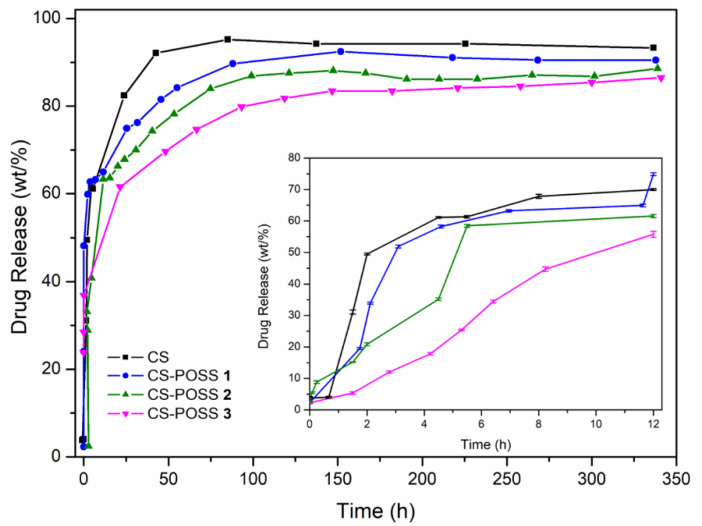
Ketoprofen release from CS and CS-POSS hydrogels at different POSS concentrations at 37 °C in PBS (pH 7.4). Data in the inset represent mean and standard deviation of three experiments in the first 12 h (SD ≤ 0.45).

The drug release behavior was investigated for the hydrogels obtained from CS and CS-POSS at different POSS concentrations, in order to study the effect of the presence of the silica cage on the drug release properties of CS-based matrices. The study was performed at the temperature of 37 °C, in phosphate buffer solution (pH 7.4) using the dialysis bag diffusion technique. The amount of drug released, evaluated in triplicate, was quantified by UV-Vis absorption spectra by measuring the absorbance of the drug at 260 nm, referring to a calibration curve recorded under the same conditions (Figure 5).

The hydrogel obtained from CS shows a release of approximately 48% of the drug in 1 h, and reaches 100% of the released drug in 1 day. For the CS-POSS-based hydrogels, the release curves also show a rapid drug release (about 37%) in the first hour (see inset in Figure 5), but a more controlled drug release in the next 2 days, reaching a plateau in the range of 79–89% of loaded ketoprofen. Furthermore, by increasing the concentration of POSS, the hybrids release the drug even more slowly, reaching CS-POSS **1**, CS-POSS **2**, and CS-POSS **3** percentages of drug release of 91%, 89%, and 86%, respectively, after 2 weeks. The results of this study suggest the presence of specific intermolecular interactions between the POSS silica cage and ketoprofen molecule, as also observed in a previous study [27]. In fact, by increasing the amount of POSS, a greater drug retention is observed in the hybrids that allow a more delayed release. The reported drug release behavior could open up new possibilities for the sustained release of drugs trapped within the polymer matrix during its biodegradation.

### 3.3. Biocompatibility Assessment of CS and CS-POSS Hydrogels

**Figure 6:**
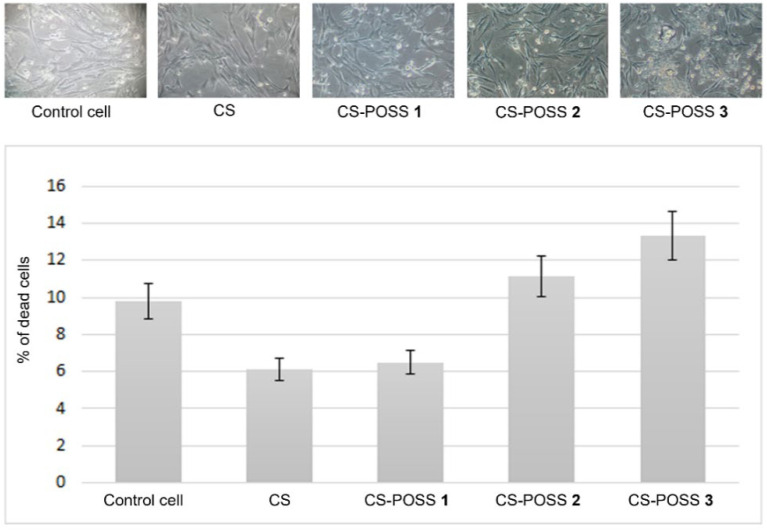
Results of MTT test for the assessment of the assayed hydrogel biocompatibility in hFOB 1.19. Each value represents the mean (±SD) of the percentage of dead cells in the experiments made in triplicate.

Human fetal osteoblastic cells (hFOB 1.19) were utilized as in vitro models to evaluate the cytotoxicity and biocompatibility of the synthesized CS-POSS-based hybrids. All samples highlighted a negligible cytotoxicity and, at the highest concentration of POSS (1.5 wt% with respect to CS), the percentage of dead cells was 13.3 vs. 9.8 detected in the control cells (*p* ns). As shown in Figure 6, both the hydrogel without POSS and the one with the lowest dose of POSS (0.5 wt% with respect to CS) even reduced cell mortality, highlighting the greater biocompatibility of this substrate when compared to the plastic materials used for cell cultures. The coating of the wells, even when partial, would seem to create a physiologically more suitable microenvironment for the cellular model used. Certainly, this is attributable to the high biocompatibility of CS, whose features are only partially neutralized by the presence of the POSS. In the hybrid material, the presence of POSS up to a concentration equal to 1% determines a cell mortality superimposable to the control cells (11.1 vs. 9.8%).

**Figure 7:**
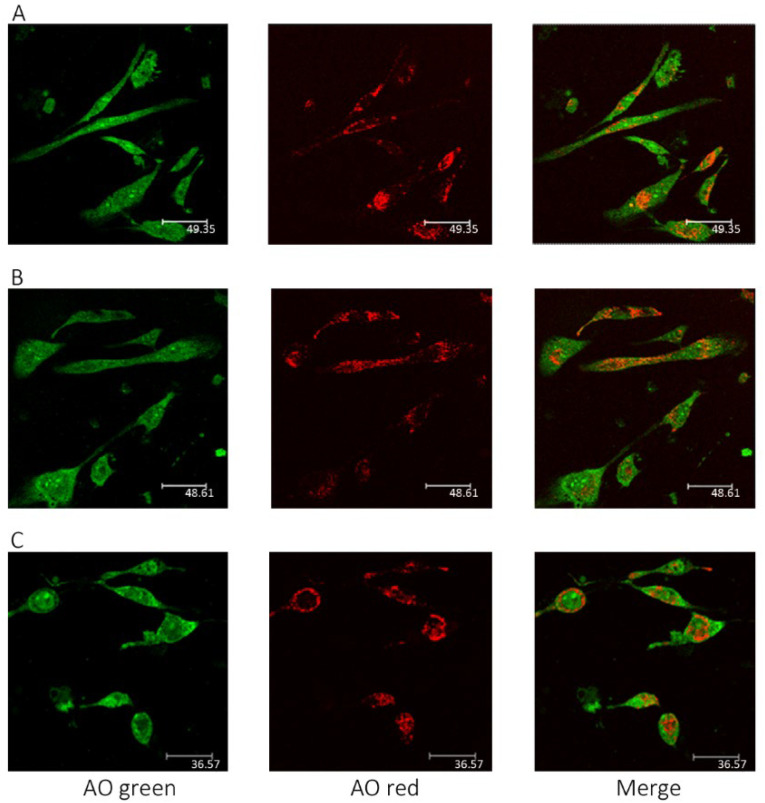
CLSM images of hFOB 1.19 cells labelled with the metachromatic fluorophore AO. (**A**) Control cells; (**B**) osteoblastic cells grown in the presence of CS hydrogel; (**C**) osteoblastic cells grown in presence of CS hydrogel with 1.5 wt% of POSS. In acidic compartment of cytosol (intact lysosomes and mature endosomes; i.e., phagolysosomes), the fluorophore is sequestered thanks to the proton pump and emits a red fluorescence, while it emits green florescence in the absence of a low pH. The cells are morphologically analogous in (**A**–**C**).

The results were also confirmed by microscopic observations using the AO probe. The CLSM images reported in Figure 7 clearly show that the cells grown in the presence of the hydrogels are morphologically similar to the controls.

## 4. Conclusions

The favorable combination of chitosan with POSS molecules was investigated for the development of hybrid hydrogels for bone tissue regeneration. The effect of different POSS concentrations within the CS matrix was evaluated by investigating the chemical and physical properties of the composites and the rheological, drug release, and in vitro biological properties of the final hydrogel composites.

The results of this study highlighted the beneficial effect of POSS molecules in improving the drug release properties and the rheological behavior of the synthesized hybrid materials. These improved properties match the biological features of the hybrids, since the in vitro biological tests performed on human fetal osteoblastic cells (hFOB 1.19) culture demonstrated their great biocompatibility at different POSS concentrations.

The combination of CS and POSS, by exploiting different conjugation procedures and different CS/POSS ratios, will represent, of course, a future research topic for the development of biocompatible and biomimetic engineered scaffolds for drug delivery, and to guide the regeneration of bone tissues.

## Figures and Tables

**Table 1:** Sample code and rheological data at two frequencies.

Sample Code	Freq. 0.1 rad/s		Freq. 10 rad/s	
	G′ (Pa)	η* (Pa*s)	G′ (Pa)	η* (Pa*s)
CS	118,183	1,197,866	148,204	12,744
CS-POSS **1**	33,082	304,315	46,645	4811
CS-POSS **2**	3684	50,983	9642	595
CS-POSS **3**	478	5132	457	45

## Data Availability

Not applicable.
